# A paradigm shift: The mitoproteomes of procyclic and bloodstream *Trypanosoma brucei* are comparably complex

**DOI:** 10.1371/journal.ppat.1006679

**Published:** 2017-12-21

**Authors:** Alena Zíková, Zdeněk Verner, Anna Nenarokova, Paul A. M. Michels, Julius Lukeš

**Affiliations:** 1 Institute of Parasitology, Biology Centre, Czech Academy of Sciences, České Budějovice, Czech Republic; 2 Faculty of Science, University of South Bohemia, České Budějovice, Czech Republic; 3 Faculty of Sciences, Charles University, Prague, Czech Republic; 4 Centre for Immunity, Infection and Evolution, The University of Edinburgh, Edinburgh, United Kingdom; University of Wisconsin Medical School, UNITED STATES

## Metabolic adaptation during *Trypanosoma brucei*’s life cycle

*Trypanosoma brucei* is a parasitic protist that causes significant health burden in sub-Saharan countries endemic for the tsetse fly (*Glossina* spp.). During the bloodmeal of this insect vector, the flagellate is transmitted to a variety of mammals, including humans, in which *T*. *brucei* subs. *gambiense* and *T*. *brucei* subs. *rhodesiense* cause human African trypanosomiasis. During its life cycle, *T*. *brucei* encounters and adapts to very diverse environments that differ in available nutrients. In the mammalian host, it exists in two major forms: the replicating long-slender bloodstream form (LS-BSF) and the nondividing short-stumpy bloodstream form (SS-BSF), the latter being pre-adapted to infect the insect vector [1]. While the BSF flagellates primarily colonize the mammalian bloodstream and utilize the plentiful glucose for their energy needs, they can also be found in the cerebrospinal fluid and in extracellular spaces of several tissues, including the brain, adipose tissue, and skin [2,3]. In the insect vector, trypanosomes occur in three major forms occupying different locations within the fly: the procyclic form (PCF) resides in the midgut and proventriculus, while epimastigotes and metacyclic trypomastigotes are found in the salivary glands. During the fly’s bloodmeal, the latter form infects the mammalian host. All three forms experience the glucose-poor and amino acid–rich environment within the insect host. These drastic environmental changes encountered by *T*. *brucei* during its development require significant morphological and metabolic changes and adaptations [4,5].

**Fig 1:**
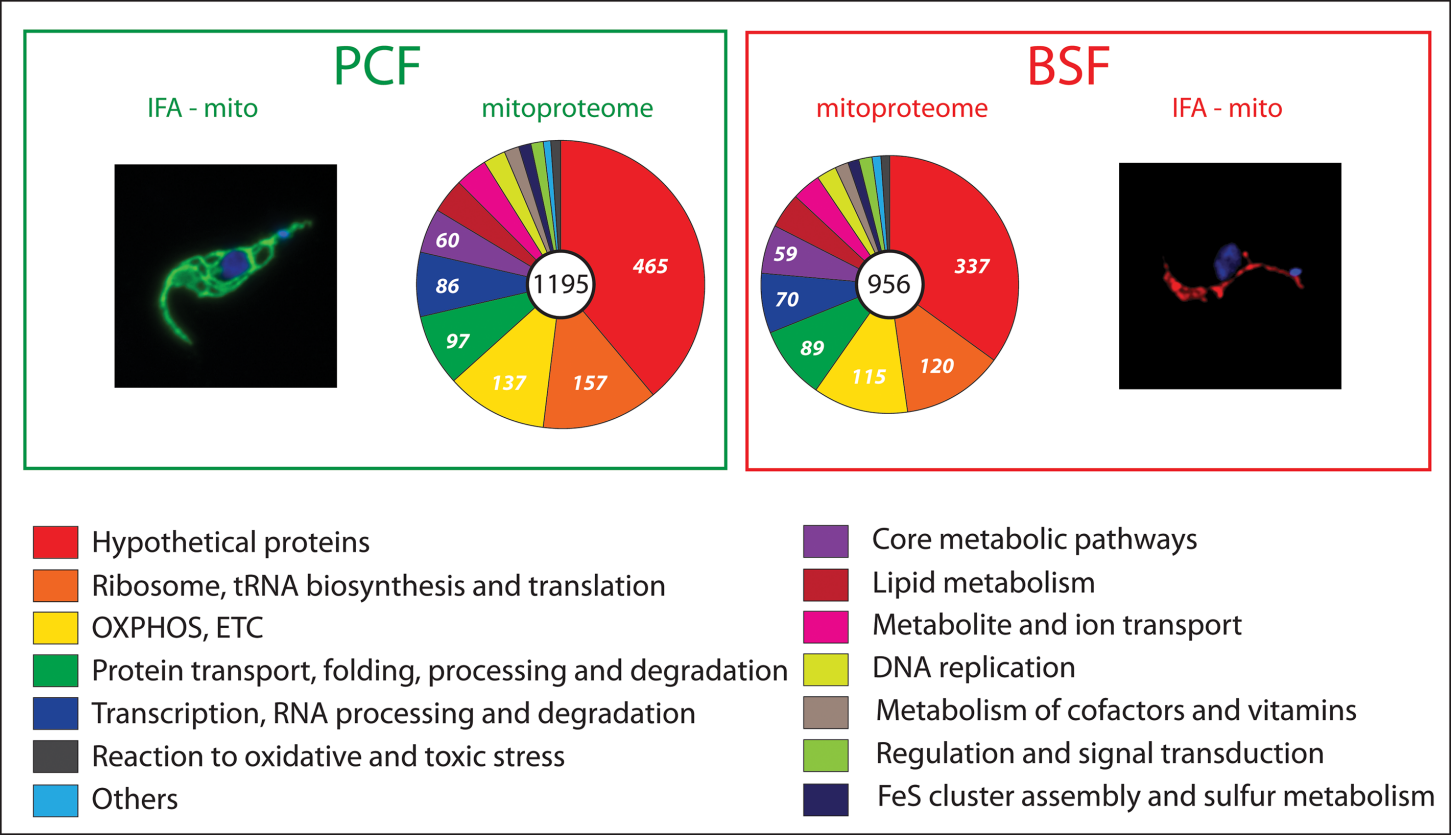
Pie charts showing distribution of mass spectrometry–identified mitochondrial proteins in PCF (left) and BSF (right) trypanosomes in terms of molecular functions. A total number of 1,195 and 956 proteins were assigned to PCF and BSF mitoproteome, respectively. Different colors show different metabolic pathways and categories. See also S1 Table. IFA-mito in PCF (left) and in BSF cell (right). BSF, bloodstream form; Hsp70, heat shock protein 70; IFA-mito, immunofluorescence analysis of a mitochondrial Hsp70; mitoproteome, mitochondrial proteome; PCF, procyclic form.

The seminal work of Keith Vickerman led to the widely accepted model of a highly reduced mitochondrial metabolism in the BSF [6,7]. Its single mitochondrion is incapable of oxidative phosphorylation, and the active electron transport chain (ETC) is minimized to an alternative pathway composed of glycerol-3-phosphate dehydrogenase (Gly-3-PDH) and the so-called trypanosome alternative oxidase (AOX), which are linked to each other via a ubiquinol/ubiquinone pool [8]. The cytochrome-containing ETC is absent, and the mitochondrial transmembrane proton gradient is generated by the reverse activity of the F_o_F_1_-ATP synthase complex at the expense of ATP [9–11]. The proton gradient across the mitochondrial inner membrane is essential for protein import and transport of metabolites and ions so that vital mitochondrial processes such as Fe-S cluster assembly [12], RNA editing and processing [13,14], and cellular Ca^2+^ homeostasis are maintained [15,16]. The seemingly simplified biochemical composition of the BSF organelle is underlined by its tube-shaped cristae-poor morphology, which is in striking contrast to the extensively reticulated cristae-rich mitochondrion of the PCF flagellates (Fig 1).

Because no indications have been obtained yet for the presence of a mitochondrial ATP-producing system in the BSF, the entire cellular ATP pool is considered to be generated solely by highly active glycolysis [17]. The glycolytic pathway in trypanosomes is unique in the sense of sequestration of most of its enzymes within peroxisome-like organelles called glycosomes [18]. Because the glycosomal membrane is impermeable to large solutes like NAD(H), the essential reoxidation of glycolytically produced intraglycosomal NADH occurs by a shuttle mechanism involving the oxidation of glycerol 3-phosphate to dihydroxyacetone phosphate by the mitochondrial Gly-3-PDH [8].

Classical metabolic studies performed with trypanosomes purified from the blood of infected rodents or with in vitro–cultured BSF supported the original hypothesis of a drastically simplified mitochondrial metabolism because under aerobic conditions, glucose is almost completely catabolized to pyruvate that is excreted from the cells, indicating no need for the mitochondrial enzymes of the tricarboxylic acid cycle. In the absence of oxygen or when AOX is chemically inhibited, glycerol 3-phosphate is converted into glycerol that is produced in a 1:1 ratio with pyruvate [19,20]. Occasionally, the production of small amounts of other compounds such as acetate, succinate, and alanine has been reported; however, these products were instead attributed to the presence of a minor fraction of SS-BSF, a life cycle stage possessing a more elaborated metabolism, with some traits characteristic of the metabolically complex PCF [21].

In preparation for differentiation into PCF, the SS-BSF up-regulates a subset of mitochondrial and other proteins [21]. Moreover, these cells are metabolically active, motile, regulate their internal pH [22], and excrete end products of glucose metabolism in ratios different than the LS-BSF and PCF cells [21]. Differentiation of LS-BSF into SS-BSF is triggered by the stumpy-inducing factor, and only pleiomorphic strains (e.g., AnTat 1.1) are able to sense this/these yet-to-be-identified molecule(s) [23]. Extended passaging of pleiomorphic parasites in in vitro cultures or by syringe between laboratory animals leads to the loss of responsiveness to the stumpy-inducing factor and thus a failure to differentiate into SS-BSF. Consequently, such strains (e.g., Lister 427) are called monomorphic, i.e., they exist only as a single form [24].

**Fig 2:**
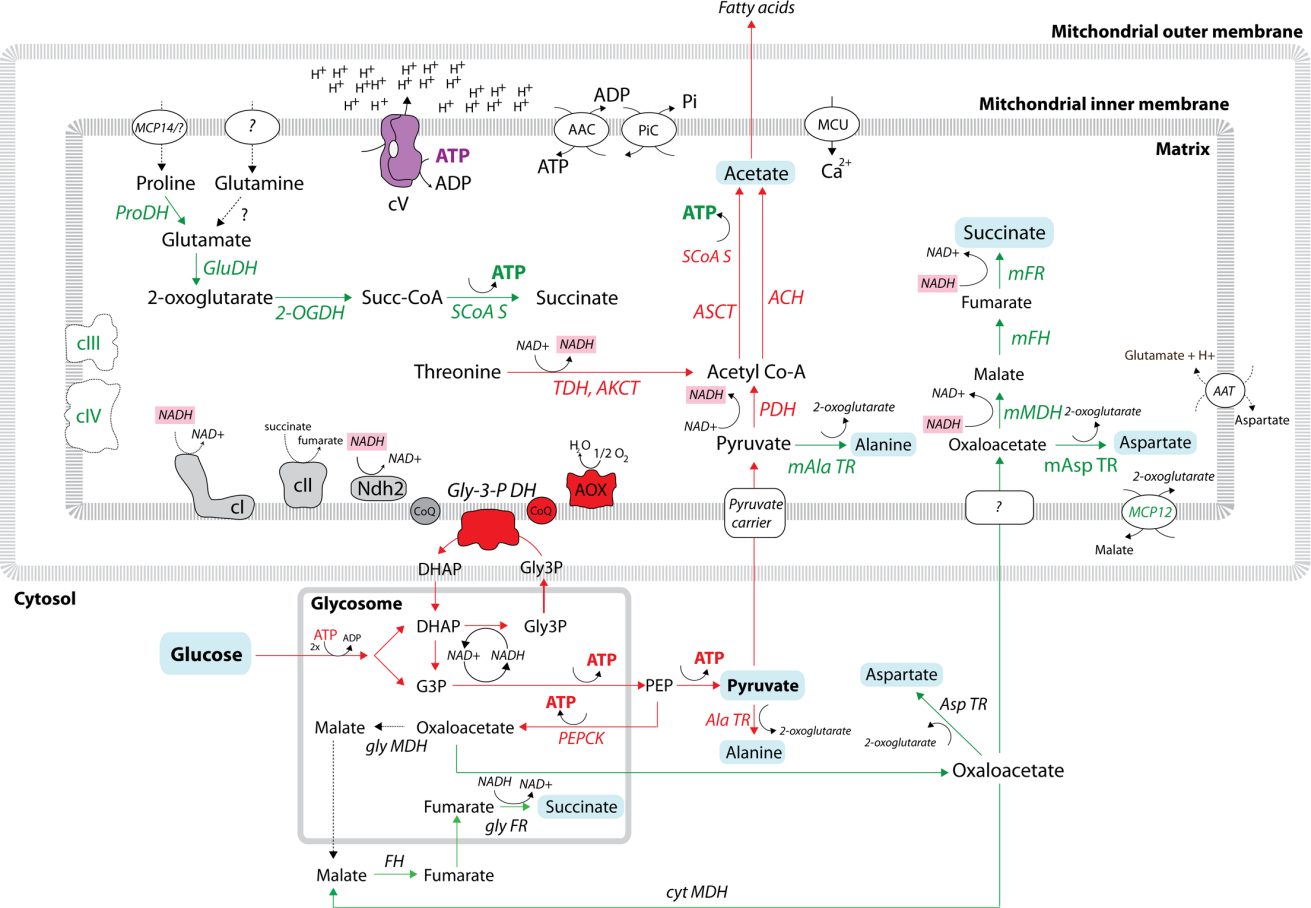
Schematic representation of carbon source metabolism in the bloodstream form of *T*. *brucei*. Red arrows represent enzymatic steps that were experimentally shown to be active in BSF. Green arrows represent enzymatic steps that might be active in BSF because the enzymes (in green) were identified in BSF proteomic data. Glucose-derived metabolites (acetate, pyruvate, succinate, alanine, aspartate) are on a blue background. NADH molecules are on a pink background. Dashed arrows indicate enzymatic steps for which no experimental proof exists. The glycosomal and mitochondrial compartments are indicated. 2-OGDH, 2-oxoglutarate dehydrogenase; AAC, ADP/ATP carrier; AAT, amino acid transporter; ACH, acetyl-CoA thioesterase; AKCT, 2-amino-3-ketobutyrate coenzyme A ligase; Ala TR, alanine transaminase; AOX, alternative oxidase; ASCT, acetate:succinate CoA-transferase; Asp TR, aspartate transaminase; BSF, bloodstream form; cI, complex I (NADH:ubiquinone oxidoreductase); cII, complex II (succinate dehydrogenase); cIII, complex III (cytochrome bc1 complex); cIV, complex IV (cytochrome c oxidase); cV, complex V (F_o_F_1_ ATPase); cyt, cytosolic; DHAP, dihydroxyacetone phosphate; FH, fumarate hydratase (i.e., fumarase); FR, fumarate reductase; G3P, glyceraldehyde 3-phosphate; GluDH, glutamate dehydrogenase; gly, glycosomal; Gly3P, glycerol 3-phosphate; Gly-3-PDH, glycerol-3-phosphate dehydrogenase; m, mitochondrial; MDH, malate dehydrogenase; PDH, pyruvate dehydrogenase; PEP, phosphoenolpyruvate; PEPCK, phosphoenolpyruvate carboxykinase; PiC, phosphate carrier; ProDH, proline dehydrogenase.

Interestingly, recent analyses employing the monomorphic LS-BSF strain Lister 427 showed that, in addition to pyruvate, appreciable amounts of other carbon products (i.e., alanine, acetate, and succinate) are excreted into the cultivation medium [25], implying a need not only for cytosolic and glycosomal but also for mitochondrial enzymes thus far considered to be absent (Fig 2). An additional metabolomics study involving heavy-atom isotope-labeled glucose determined that a substantial fraction of succinate, as well as metabolic intermediates such as malate and fumarate, are glucose-derived and originate from phosphoenolpyruvate via oxaloacetate. Importantly, phosphoenolpyruvate carboxykinase, a glycosomal enzyme responsible for this conversion, is essential for the BSF parasites [26]. Moreover, the majority of excreted alanine and acetate is also derived from glucose. Alanine is most likely produced from pyruvate by the transamination reaction of alanine aminotransferase, a potentially essential enzyme [27], while glucose-derived acetate is produced from pyruvate by the mitochondrial pyruvate dehydrogenase (PDH) complex and additional subsequent enzymatic steps. A fraction of the acetate produced this way is exported to the cytosol for the de novo synthesis of fatty acids, which is an essential process (Fig 2) [25]. In addition to glucose, the BSF seems to uptake and metabolize amino acids such as cysteine, glutamine, phenylalanine, tryptophan, and threonine [28], implying the existence of an unexpectedly complex metabolic network in their mitochondrion.

## The BSF mitoproteome

To map the BSF mitochondrial proteome (mitoproteome), we first used the available mass spectrometry data of purified PCF mitochondria [29–37] in order to assemble a comprehensive list of mitochondrial proteins. Next, we asked how many of these proteins were identified in any mass spectrometry data obtained from BSF cells [38–43]. To our surprise, out of 1,195 constituents of the PCF mitoproteome, 956 were also identified in at least one study of the BSF, suggesting that, when qualitatively measured, the corresponding mitoproteome is reduced by only approximately 20% (Fig 1; S1 Table). The surprisingly high, approximately 80% overlap with the PCF mitoproteome might also be a consequence of the heterogeneity of the examined BSF populations. The heterogeneity may be related to the experimental protocols, the environmental variations (cells grown in vivo versus in vitro), or variations within the cell cycle (e.g., ATP requirements vary between different cell cycle stages) as well as to the form type (monomorphic versus pleiomorphic). Indeed, some authors analyzed monomorphic strains grown in vitro [40,41], and others examined the pleiomorphic AnTat 1.1 strain grown either in immunosuppressed rats [43] or in vitro (S1 Table) [38]. Therefore, some LS-BSF cells may have a mitochondrion that is close to the “classical” version, while a subset of these flagellates may express an extended mitoproteome. However, no apparent differences were detected between the mitoproteomes from the pleiomorphic and monomorphic BSF cells, suggesting that, regardless of their status, a surprisingly large repertoire of mitochondrial proteins is expressed in the BSF stage.

All proteins were then organized into groups based on their Kyoto Encyclopedia of Genes and Genomes (KEGG) annotations. No striking qualitative differences were observed in the categories “oxidative phosphorylation” and “core metabolic pathways” comprising many enzymes involved in the carbon, amino acid, and energy metabolism (Fig 1, S1 Table). Typical examples are components of the tricarboxylic acid cycle and subunits of the ETC complexes, most prominently of respiratory complexes III and IV (Fig 2, S1 Table). Nonetheless, when quantitative information was available, these proteins were often present in much lower amounts than in the PCF. While some of these proteins may not perform their expected function(s) under BSF steady state growth conditions, this finding strongly suggests that the parasite is capable of swift alterations or adjustments of its metabolism in response to various environments and differentiation cues. This ability can be exploited during environmental changes, for example when the LS-BSF migrates from the peripheral blood circulation to other extravascular spaces (e.g., in adipose tissue, spinal and cerebral fluids) and during the differentiation to SS-BSF. Therefore, the BSF trypanosomes may uptake different substrates from the available nutrients according to their immediate needs and metabolize them via a variety of pathways.

## Complex metabolic pathways in the BSF mitochondrion: Does presence equal activity?

The current metabolic model for BSF excludes a role of the mitochondrion in the ATP production by either oxidative or substrate-level phosphorylation [44]. In contrast to this premise, succinyl-CoA synthase (SCoAS), an enzyme responsible for substrate-level phosphorylation of ADP to ATP, has been detected in BSF cells, and more importantly, its RNA interference (RNAi)–mediated silencing produced a severe growth phenotype [45]. This enzyme can be involved in two ATP-producing pathways. The first one includes activity of 2-oxoglutarate dehydrogenase (2-OGDH) producing succinyl-CoA from 2-oxoglutarate that originates from amino acids such as proline and glutamine or can result from transamination reactions by mitochondrial alanine and aspartate transaminases (Fig 2). While all the enzymes involved in these reactions were detected in the LS-BSF mitoproteome (Fig 2 and S1 Table), the activity of 2-OGDH remains contradictory because some authors failed to detect it in the pleiomorphic cells [46], while others recorded its low activity in culture-adapted monomorphic LS-BSF cells [47]. Puzzlingly, the 2-OGDH subunits E1 and E2 were shown to be essential in BSF not because of their role in carbon metabolism but rather due to their moonlighting roles in glycosomes and mitochondrial DNA maintenance [46,48]. However, in an untargeted metabolomics study using isotope-labeled glucose, up to 30% of excreted succinate remained unlabeled, supporting its nonglucose origin [26] and making the occurrence of this substrate-level phosphorylation reaction even more plausible (Fig 2).

The second phosphorylation pathway includes the acetate:succinate CoA transferase/SCoAS cycle that contributes to acetate production in the BSF mitochondrion. A substrate for this reaction—acetyl-CoA—is produced by PDH, an enzymatic complex that is present and active in the BSF mitochondrion [15,25]. Moreover, PDH was shown to be indispensable for BSF cells but only in the absence of threonine because under these artificial conditions, PDH was the only system supplying acetyl-CoA for the essential acetate production [25]. Nonetheless, the mitochondrial pyruvate transporter was demonstrated to be essential for BSF in vivo, supporting PDH’s vital role for the parasite [49]. These results imply that the BSF mitochondrion may become an ATP producer under certain conditions, perhaps just for intramitochondrial needs (Fig 2).

The presence and potential activity of the aforementioned dehydrogenases that produce NADH within the mitochondrion imply that the organelle would require reoxidation of this cofactor. Several possible scenarios for such a capacity can be deduced from the available data (S1 Table). Although complex I (NADH:ubiquinone oxidoreductase) was shown to be neither essential for BSF nor contributing to the observed NADH:ubiquinone oxidoreductase activity [50], it is still assembled in the BSF mitochondrion and may participate in NADH reoxidation under certain conditions. Reoxidation of reduced NADH molecules can also be achieved by the activity of the alternative dehydrogenase 2 (Ndh2), an enzyme shown to be important but not essential for maintaining the mitochondrial redox balance [51]. Last but not least, another scenario includes the activities of the mitochondrial malate dehydrogenase, fumarate hydratase (i.e., fumarase), and NADH-dependent fumarate reductase, with all three being present in the BSF mitoproteome (Fig 2, S1 Table). These enzymes reduce glucose-derived oxaloacetate via malate and fumarate to succinate. Indeed, 3-carbon–labeled succinate was identified in an untargeted metabolomics study, implying that this pathway might be active [26]. Still, it should be noted that it is so far impossible to discriminate between the mitochondrial, glycosomal, and cytosolic derivations of this metabolite and that only a systematic deletion of the corresponding enzymatic isoforms followed by metabolomics would illuminate the cellular compartment in which this glucose-derived succinate is produced. To sum up, the collective activity of the aforementioned reoxidation enzymes is most likely responsible for the mitochondrial NADH regeneration. Possibly, RNAi silencing of the mitochondrial malate dehydrogenase and fumarate reductase in the background of complex I and Ndh2 null mutants would shed light on the quantitative role of each of these enzymes in mitochondrial NADH reoxidation.

The possible occurrence of the mitochondrial substrate-level phosphorylation reactions raises an interesting question regarding the mitochondrial bioenergetics of BSF and questions the origin of ATP that is needed by mitochondrial F_o_F_1_ ATPase in order to maintain the mitochondrial membrane potential. The classical model presumes that ATP is imported into the organelle via the activity of the ATP/ADP carrier [52,53]. However, the available data—such as low sensitivity of BSF to treatment with bongkreic acid, an inhibitor of this carrier—raise some doubts about this assertion. Interestingly, the BSF does not respire when the mitochondrial transmembrane proton gradient is dissipated upon treatment with the F_o_F_1_ ATPase inhibitor oligomycin or by addition of carbonyl cyanide-*4*-(trifluoromethoxy)phenylhydrazone (FCCP). However, when treated with bongkreic acid, which should halt the activity of F_o_F_1_ ATPase by restraining its substrate, the parasite consumes oxygen at the same rate as untreated cells [54,55]. On one hand, it is possible that the mitochondrial inner membrane harbors another ATP/ADP carrier; on the other hand, it is a plausible speculation that, when specific conditions emerge, the BSF mitochondrion has the capacity to employ its complex enzymatic network to produce ATP by substrate-level phosphorylation to power the F_o_F_1_ ATPase.

## Concluding remarks

Combined, the available data reveal that the metabolic flexibility and adaptability of the BSF mitochondrion are much larger than appreciated so far. Mitochondrial metabolism appears to be controlled at various levels; a developmental program seems to be a major contributor, but recent advances in the field suggest that other cues may also play a role through fine-tuning mechanisms. However, the triggers and signaling pathways of these mechanisms remain to be identified. Furthermore, it should be realized that almost all metabolic studies have been performed with strains well adapted to laboratory conditions. While the proteomic data do not show any significant differences between the monomorphic and pleiomorphic strains, future work combining proteomics and metabolomics with functional genomics should be extended to the mitochondrion of trypanosomes isolated not only from blood but also from other tissues to determine whether their metabolism is tissue specific and, if so, what is/are the mechanism(s) that control(s) the changes. Therefore, the virtually unexplored array of pathways and enzymes begs for attention because it may have important implications for drug target identification and future novel chemotherapeutics. Moreover, a decreased morphological complexity, which is apparently not reflected in metabolic complexity, is an interesting and novel phenomenon that can now be efficiently addressed with emerging, increasingly sensitive methods.

## Supporting information

S1 Table List of mitochondrial proteins that were identified in proteomic analysis of PCF cells (columns F, G, and H) and of BSF cells (columns I, J, K, L, M, and N). The column color coding is green for PCF, dark grey for monomorphic BSF, and light gray for pleiomorphic BSF cells.
1, identified; 0, not identified; BSF, bloodstream form; PCF, procyclic form.
(XLSX)
